# Isolated splenic peliosis: a case report 

**DOI:** 10.1186/s13256-023-03929-7

**Published:** 2023-06-30

**Authors:** Bahaa Nassr, Wael Abdu Hassan, Hasan Nassr, Abdullah Allouzi, Mohammed Al-Shebly

**Affiliations:** 1 Department of General Surgery, Aster Sanad Hospital, Riyadh, Saudi Arabia; 2 Department of Basic Sciences, Sulaiman Al Rajhi Colleges, PO Box 777, Al Bukayriyah, 51941 Saudi Arabia; 3 College of Medicine, Sulaiman Al-Rajhi University, Al Bukayriyah, Al-Qassim Saudi Arabia; 4 Department of Pathology, Faculty of Medicine, Suez Canal University, Ismailia, Egypt

**Keywords:** Peliosis, Splenic peliosis, Spontaneous splenic rupture, Spleen, Splenectomy, Case report

## Abstract

**Background:**

Peliosis is a rare condition with anatomopathological characteristics that affect the liver. However, splenic peliosis is even more unique and rare. Patients with such abnormality usually exhibit no symptoms. Moreover, this is a lethal condition due to the high probability of splenic rupture accompanied by shock.

**Case presentation:**

We present a case of a 29-year-old Arab female who was admitted to the hospital with severe upper abdominal pain that started 1 week from the date of admission, associated with nausea, anorexia, low-grade fever, and vomiting, with no past medical history or comorbidities. A computerized tomography scan with contrast showed intraperitoneal free fluid along with multiple hypodense splenic cysts. Hence, an emergent exploratory laparotomy with splenectomy was performed. Splenic peliosis was confirmed by the histopathological examination.

**Conclusion:**

Further investigations are warranted if peliosis is confirmed in one organ, for example, the liver, to detect its presence in any other potential organs that can be affected by peliosis. Splenic peliosis is extraordinarily rare. Furthermore, such a disease has no established management plan. Definitive treatment is surgical. Many aspects of splenic peliosis remain puzzling requiring more research in the near future.

## Background

In 1861, Wagner first used the Greek word “pelios,” which means dusky or purple [1]. Peliosis, typically affecting the liver, is characterized by cyst-like blood-filled cavities that form within the parenchyma [2]. Isolated splenic peliosis is considered to be extremely rare. However, peliosis of the spleen has been associated with chronic disorders, including cancer, infections, and steroid intake [1]. It is one of the diseases that progresses silently, and usually, the patients display no symptoms [1]. Hemorrhagic cysts may burst spontaneously or rupture due to trauma, which will lead to the progression of a life-threatening hemorrhagic shock; hence, fatal outcomes may develop [1].

In this paper, we are describing a case of an adult female patient with spontaneous splenic rupture associated with an isolated incidence of splenic peliosis. Pathological, radiological, and surgical findings along with the management plan for the patient are discussed.

## Case presentation

A previously healthy 29-year-old Arab female was brought by her father to the emergency department of Aster Sanad Hospital for severe upper abdominal pain. The pain started 1 week previously and progressed, becoming severe at the time of admission. It was radiating to the left shoulder and was associated with nausea, anorexia, low-grade fever, and vomiting. Moreover, the pain was reduced upon lying flat and exacerbated by movement. The patient mentioned that she went to see her family in a nearby village where she was in indirect contact with domestic animals. Past medical history and systemic review analysis were unremarkable. Normal saline 0.9% NaCl (500 mL) and intravenous acetaminophen 1 g were given to the patient in the emergency room.

Initial general physical examination showed hemodynamic stability as per the following: pulse rate 97 beats/minute, respiratory rate 18 breaths/minute, blood pressure 113/95 mmHg, oxygen saturation 96%, and temperature 38 °C. On admission, the Glasgow Coma Scale (GCS) score was 13. Abdominal examination showed generalized abdominal tenderness, muscle guarding, and rigidity in the upper part of the abdomen, eliciting the findings of acute peritonitis. Hypoactive bowel sounds were heard on auscultation. Cardiopulmonary and peripheral vascular examinations were unremarkable.

**Fig. 1:**
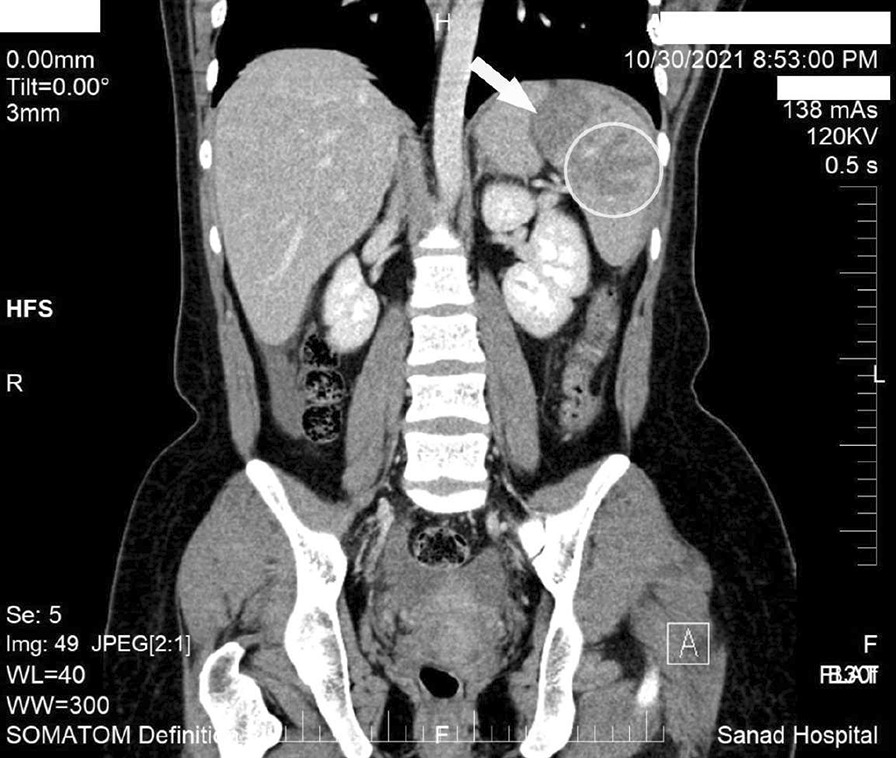
Abdominal computerized tomography scan with contrast. Coronal section of the computerized tomography scan shows hypodense finger-like projections on the spleen, as a result of the dilated sinusoidal channels (white circle). One of the ruptured splenic cysts is shown (white arrow)

An emergency computerized tomography (CT) scan with contrast was ordered upon admission (Fig. 1). Evidence of multiple variable-sided hypodense lesions was seen scattered in the spleen. Evidence of mild to moderate intraperitoneal hyperdense free fluid collection was seen at the perisplenic, perihepatic, lower abdominal, and pelvic regions; probably hemorrhagic fluid. Normal CT appearance of the intra- and extrahepatic biliary radicles was observed. Normal appearance of the pancreas was observed, with preserved surrounding fat planes. Both kidneys were of normal size. No masses, stones, or back pressure changes were detected. Normal appearance of both adrenal glands, and all of the different segments of the small and large bowel loops, was noted.

Complete blood count (CBC), coagulation profile, and creatinine tests were ordered upon admission. The CBC showed the following: red blood cell (RBC) count 3.67 × 10^6^/UL (low), hemoglobin 9.8 g/dL (low), hematocrit 29.5% (low), red cell distribution width–coefficient of variation (RDW–CV) 16.3% (high), and red cell distribution width–standard deviation (RDW-SD) 48.8 FL (high). Coagulation profile results were normal. Creatinine level was 41 µmol/L (low).

**Fig. 2:**
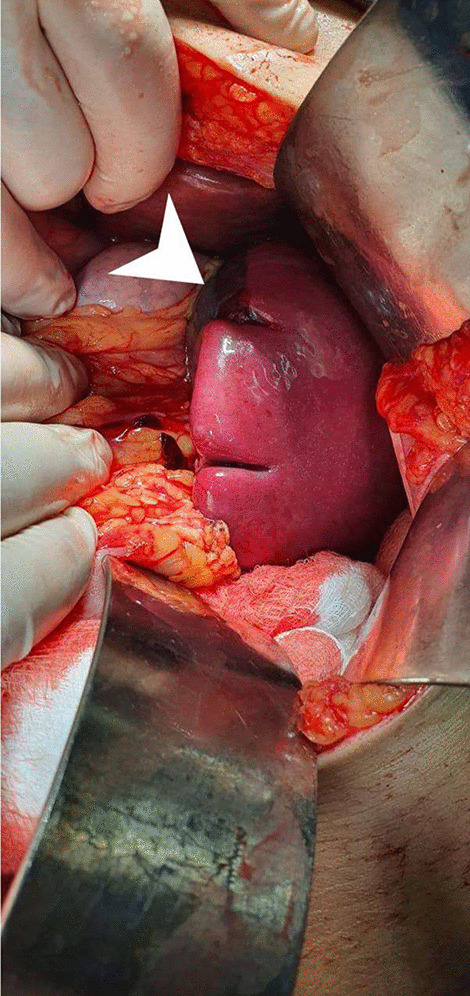
Explorative laparotomy showing ruptured cyst. Explorative laparotomy shows ruptured splenic cysts (white arrowhead) after suctioning the blood covering the entire abdomen

An emergent explorative laparotomy with splenectomy was performed. Before the surgery, pneumo-meningococcal and flu vaccine was given. Under general anesthesia and aseptic technique, the skin was draped. A midline skin incision was done. Upon exploration, blood was found to be all over the abdomen. There were ruptured bleeding cysts in the spleen, as shown in Fig. 2. Hence, ligation of splenic vessels was done followed by the removal of the spleen. Omentoplasty in the cyst space was performed. A drain was inserted in the pelvis and fixed. Finally, the dissected spleen was sent for pathological examination.

Pathological examination of the splenectomy specimen revealed a spleen weighing 900 g and measuring 18 cm × 4 cm × 6 cm, with an intact capsule, smooth surface with focal laceration, and preserved hilum. Cut section of the spleen revealed dark brown homogeneous splenic tissue with no nodules or masses detected.

**Fig. 3:**
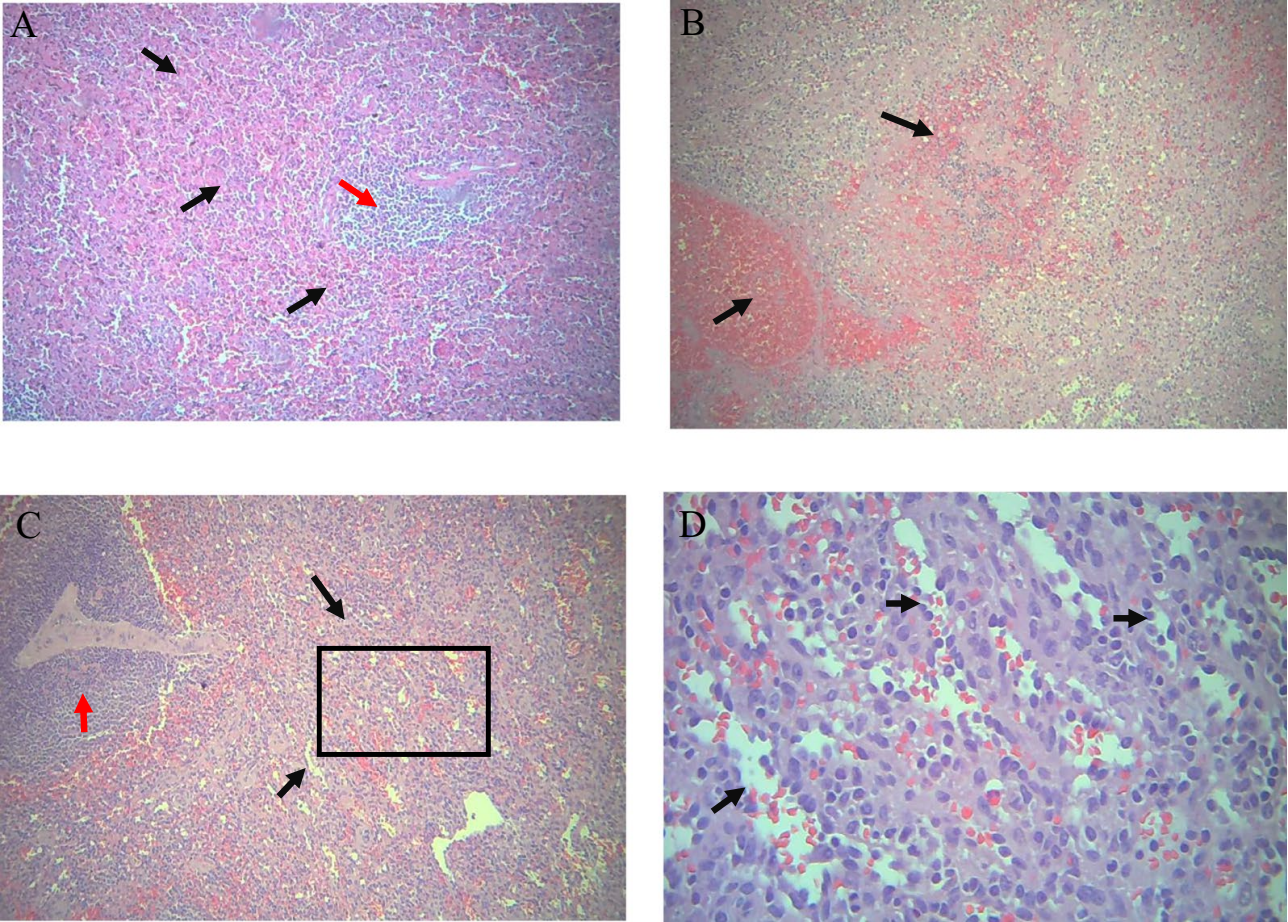
Histopathological features of splenic lesions. **A** Splenic tissue showed marked congestion in splenic cords with dilated sinusoids (black arrows), with atrophy of lymphoid follicles (red arrows) [hematoxylin and eosin (H&E), ×100]. **B** Many foci in spleen showed proliferated cystic lesions (black arrows) containing blood and fibrinous debris (H&E, ×100). **C** Other foci in spleen showed proliferation of sinusoid-like channels within red pulp (Black arrows within rectangular black area) with atrophied lymphoid follicles (Red arrow) (H&E, ×100). **D** Higher magnification of the previous figure showing that these proliferated sinusoid-like channels are lined with plump endothelial cells (black arrows) and filled with blood (H&E, ×400)

Microscopic examination of sections prepared from the submitted spleen (Fig. 3) revealed splenic tissue with marked congestion in cords, with dilated sinusoids, and packed with RBCs. There were numerous cystic lesions lined by fibrous tissue with no identified epithelial or endothelial cell lining, and containing blood and fibrinous debris, with adjacent chronic inflammatory cell infiltrate. There were other foci in the spleen that showed proliferation of sinusoid-like channels, lined with plump endothelial cells and filled with blood. Diagnosis of congestive splenomegaly, with splenic peliosis and splenic vascular hamartoma, was established.

The patient was discharged with the following medications: amoxicillin/clavulanic acid 1 g [twice daily (BID) for 1 week], diclofenac 50 mg (every 12 hours for 1 week), paracetamol 1 g [three times daily (TID) for 1 week], pantoprazole 40 mg (once daily for 1 week), and enoxaparin 40 mg injection subcutaneously (once daily for 7 days). There were no postsurgical complications. On follow-up after 1 week from discharge, the patient was well but displayed thrombocytosis with a platelet count of 1,000,000/μL. Thus, she was prescribed low-dose aspirin of 81 mg.

## Discussion and conclusion

Whenever the term peliosis is placed within the medical scheme, the liver is unintentionally the first and only organ to be thought of having these dilated sinusoidal channels; hence, the term peliosis hepatis is what many individuals will think about and start elaborating on. However, later on it was discovered that peliosis can be present in any of the organs of the reticuloendothelial system, including the bone marrow, spleen, liver, or lymph nodes [2]. When Wagner first defined peliosis, he was defining the gross specific lesions [3]. Nevertheless, peliosis is now defined on the basis of histopathological findings.

A case of splenic peliosis is rare and usually presents secondary to peliosis hepatis. Moreover, the isolated presence of splenic peliosis is infrequent and scarce. The first reported case of splenic peliosis (also known as peliosis lienis) was published in 1978 [4]. The exact pathogenesis of peliosis is unknown. However, several etiological factors were suggested to be associated with peliosis in the spleen, including the following: intravenous drug abuse, the usage of anabolic steroids, chemotherapy, liver cirrhosis, tuberculosis, oral contraceptive pills, human immunodeficiency virus (HIV), Hodgkin’s disease, aplastic anemia, and myeloma [3–5]. However, for our patient, she had no past or current comorbidities to be intervening in her case of peliosis.

Patients with splenic peliosis are usually asymptomatic. In fact, patients with such rare conditions will not seek medical attention until they develop splenic rupture. Moreover, one literature review has shown that all the reported cases collected from 1980 till 2016 had either presented with abdominal pain or circulatory shock [6]. Thus, such findings indicate the importance of including such a rare condition in the differential diagnosis of acute abdomen, especially if the patient’s status is accompanied with established hemodynamic instability. As there are no clear published guidelines on the management of splenic peliosis, the definitive management of splenic rupture due to peliosis is emergent splenectomy, matching the treatment plan that our patient has undergone [1–4]. However, prophylactic splenectomy is still debatable if accidental radiological findings were established upon regular check-ups or unrelated complaints.

In conclusion, the condition of isolated splenic peliosis with no comorbidities is an extraordinary condition that can be fatal if no emergent actions are taken toward the patient upon sudden splenic rupture. Many aspects are not well understood or clear in the medical scheme in recent times. The scarcity of the reported cases is a prominent limiting factor for the establishment of clear management algorithms and suitable diagnostic tools. Therefore, it is essential for healthcare workers to constantly report and publish any new or different findings, cases, and rare conditions to clarify the vagueness of the challenging medical dilemmas.

## Data Availability

No datasets were generated or analyzed during the current study.
